# Aging and Bone Health in Individuals with Developmental Disabilities

**DOI:** 10.1155/2012/469235

**Published:** 2012-07-22

**Authors:** Joan Jasien, Caitlin M. Daimon, Stuart Maudsley, Bruce K. Shapiro, Bronwen Martin

**Affiliations:** ^1^Metabolism Unit, National Institute on Aging, National Institutes of Health, 251 Bayview Boulevard, Suite 100, Baltimore, MD 21224, USA; ^2^Department of Neurology and Neurodevelopment, Kennedy Krieger Institute, 801 N. Broadway, Baltimore, MD 21224, USA; ^3^Receptor Pharmacology Unit, National Institute on Aging, National Institutes of Health, 251 Bayview Boulevard, Suite 100, Baltimore, MD 21224, USA

## Abstract

Low bone mass density (BMD), a classical age-related health issue and a known health concern for fair skinned, thin, postmenopausal Caucasian women, is found to be common among individuals with developmental/intellectual disabilities (D/IDs). It is the consensus that BMD is decreased in both men and women with D/ID. Maintaining good bone health is important for this population as fractures could potentially go undetected in nonverbal individuals, leading to increased morbidity and a further loss of independence. This paper provides a comprehensive overview of bone health of adults with D/ID, their risk of fractures, and how this compares to the general aging population. We will specifically focus on the bone health of two common developmental disabilities, Down syndrome (DS) and cerebral palsy (CP), and will discuss BMD and fracture rates in these complex populations. Gaining a greater understanding of how bone health is affected in individuals with D/ID could lead to better customized treatments for these specific populations.

## 1. Introduction

Developmental disability (DD) is a group of severe chronic conditions that are attributable to an impairment in physical, cognitive, speech, language, psychological, or self-care areas that are manifested during the developmental period (younger than 22 years of age) [1]. Intellectual disability (ID) is a disability characterized by significant limitations both in intellectual functioning and in adaptive behavior, which covers many everyday social and practical skills. This disability originates before the age of 18 [2]. ID has been classified through performance on IQ tests as mild (IQ 69–55), moderate (IQ 54–40), severe (IQ 39–25) or profound (IQ <25). These cutoffs are typically based on tests with a mean score of 100 and a standard deviation of 15 and do not reflect the standard error of measurement, which is approximately 5 points. If an individual is intellectually disabled, they could also be identified as having a developmental disability. However, one can possess a developmental disability, such as a motor or language disability, and not be intellectually disabled. Down syndrome (DS) and cerebral palsy (CP) are two examples of DD. DS individuals are intellectually disabled, and approximately fifty percent of individuals with CP have an intellectual disability.

The life expectancy of the general healthy population has increased significantly over the past decades. Although many studies have investigated the aging process in the general population, relatively little attention has been paid to aging in people with developmental disability/intellectual disability (D/ID). Rigorous and robust studies that investigate the aging process in individuals with D/ID are currently lacking. The exact reasons for this disparity in aging research are unclear, but a change in society's approach to the care of individuals with D/ID has likely been a major contributing factor. Prior to the 1970s, many individuals with D/ID did not live long enough to complete rigorous aging studies and individuals with D/ID were predominantly deinstitutionalized in the United States. In 1983, Carter and Jancar examined trends in the causes of death and mortality rates in patients with “*mental handicaps*” residing in an institution in the United Kingdom between 1930 and 1980. From 1931 to 1935, the average life expectancy for males with D/ID was 14.9 years and 22.0 years for females with D/ID. Poor sanitary conditions and nutrition, lack of adequate medical care, and crowding in the institutions during that period have been attributed to this disparity in aging [3]. Tuberculosis was a major cause of death until the 1950s, and the life expectancy increased slowly over the years from 1971 to 1975, until it was approximately 49.8 years for males and 54.1 years for females with D/ID [3]. This trend of increasing longevity has also been seen in the DS population; people with DS experienced a doubling in life expectancy over a 14-year span [4]. In 1983, the average lifespan for an individual with DS was 25 years, and by 1997 it had increased to 49 years [5]. This doubling of life expectancy has been attributed to numerous factors, such as improved medical interventions during childhood (e.g., cardiac surgery), improved living environments, diets, and illness interventions such as diagnosis and treatment of hypothyroidism [6–8]. Currently, the life expectancy for many individuals with D/ID is similar to that of the general healthy population, except for adults with certain genetic/metabolic conditions and with a more severe intellectual disability. For mild to moderate developmental disabilities, life expectancy is approximately 70 years, although for DS and severe developmental disabilities it is approximately 50 years [4, 9, 10]. 

The reason for the persistent shorter lifespan for the DS population, compared to many of the other developmental disabilities, is thought to be due to an accelerated aging process, which is manifested by increased rates of cataracts, hearing loss, osteopenia, hypothyroidism, and a genetically elevated risk for developing Alzheimer's disease [11]. The population of adults with an intellectual and developmental disability aged 60 and older is projected to double from 641,860 in 2000 to 1.2 million by 2030. This could be attributed to an increasing life expectancy and aging of the “*Baby Boomer*” generation [12]. Due to this increased longevity, individuals with D/ID are confronted by many of the same chronic illnesses that affect the general aging population, but their onset may be earlier and the effects diverge in severity. Effective studies to determine this however are currently lacking, and there is a dearth of informative literature on this topic. The aim of this paper is to provide an overview of the present literature on some of the aging aspects of intellectual and developmental disabilities. We will specifically focus on a key aspect of the aging process, bone health and bone mass density (BMD), in two important and common developmental disabilities: DS and CP. First, we will provide a brief overview of bone health and fractures in the normal aging population; next, we will provide an overview of studies on bone health in adults with D/ID, including BMD, risk factors for low BMD, fracture rates, and a limited discussion of treatment studies. Lastly, bone health in the DS and CP populations will be discussed.

## 2. Bone Health in the Normal Aging Population

Osteoporosis is one of the most common conditions associated with aging in the general population [13]. Osteoporosis is a disease of bones that leads to an increased risk of fracture. In osteoporosis the BMD is reduced, bone microarchitecture deteriorates, and significant protein expression alterations in bone are apparent. Osteoporosis is defined by the World Health Organization (WHO) as a bone mineral density that is 2.5 standard deviations or more below the mean peak bone mass (average of young, healthy adults) as measured by dual-emission X-ray absorptiometry (DXA). Using DXA, two measures of bone health are typically obtained: a *T* score and a *z *score. According to the WHO definition, a *T* score is the comparison of a person's bone density with that of a healthy 30-year old of the same sex. Osteoporosis is defined by a *T* score of −4.0 to 2.5, osteopenia is −2.5 to −1.0, normal bone mass is −1.0 to 1.0, and high normal bone mass is 1.0 to 4.0. The *z* score is a comparison of a person's bone density with that of an average person of the same age and sex. Bone is being continuously turned over in distinct areas of the skeleton due to bone-forming osteoblasts and bone-resorbing osteoclasts [14, 15]. In healthy young adults, bone resorption and formation are tightly linked, thereby maintaining a steady state of bone [15]. However, during the aging process, significant bone loss occurs due to the tipping of this finely tuned equilibrium towards enhanced resorption, coupled to decreased bone formation [15, 16]. This net loss in bone mass during the aging process can ultimately lead to osteoporosis [15]. A multitude of factors are known to play a role in maintaining adequate bone health, including nutrition, lifestyle choices, genetics, and hormonal status [16]. 

A recent observational cross-sectional study analyzed risk factors in aging subjects with a recent clinical fracture [17]. Over the course of one year, men and women over fifty years of age who presented to a medical facility with a clinical fracture were invited to participate in a bone- and fall-related risk factor assessment and receive a bone density measurement. The bone-related risk factors for fracture assessment included a previous fracture after the age of fifty, a mother with a fracture history, a body weight of <60 kg, severe immobility, and the therapeutic use of glucocorticoids. The fall-related risk factors for fractures included more than one fall in the past year, the use of psychoactive drugs, a low level of activities of daily living before the current fracture, articular symptoms, impaired vision, urinary incontinence, and Parkinson's disease. This fracture risk factor assessment was based on the Dutch guidelines for the prevention of osteoporosis and falls [17]. Patients were excluded if they were receiving treatment for osteoporosis or had a pathologic fracture. This study comprised 406 women with a mean age of 68 years and 162 men with a mean age of 65 years. It was found that the prevalence of fall-related risk factors (75%, *n* = 425) and the prevalence of bone-related risk factors (53%, *n* = 299) at the time of fracture were higher than the prevalence of osteoporosis (35%, *n* = 201). Fall- and bone-related fracture risk factors were present and independent of fracture location, age, or gender. Fifty percent of the patients had an overlap between bone and fall-related risk factors. After adjusting for age, weight, and height, women with a fracture were found to more frequently have a diagnosis of osteoporosis and have a more frequent history of falls than did postmenopausal women without a fracture history. This study implies that in order to predict fractures in an aging population, knowing bone-related and fall-related risk factors could be just as important as actual BMD measurements [18]. Additionally, immobility was also found to be a significant risk factor for recurrent fractures in the normal elderly population [19]. Fractures in the normal elderly general population not only lead to pain and immobility but also mortality and institutionalization. In a study seeking to identify determinants of mortality and institutionalization after hip fractures and also hip fractures in patients at high risk of death or institutionalization after hip fracture, cognitive impairment was found to increase the chances of mortality and institutionalization [20]. In this study, male gender was found to also increase mortality risk fourfold. Patients with lower postfracture physical function had at least five times the risk of institutionalization, compared to patients with high postfracture physical function [20]. 

Osteoporosis not only affects women in the normal aging population but also men, and it can have detrimental effects. Estrogen deficiency appears to be a major factor in the pathogenesis of osteoporosis in both genders [13]. However, mortality after a hip fracture, one of the major complications of osteoporosis, is more common in men than in women. Some of the risk factors for low BMD in males that have been assessed include calcium intake, exercise, alcohol consumption, and smoking [21]. Age-related bone loss and osteoporosis generally put the elderly population at an increased risk for fractures and morbidity. However, our understanding of age-related bone loss in the normal healthy population has increased greatly in recent years and has led to better diagnoses and treatments. This is not the case for bone loss in other populations, such as those with developmental disability, and more studies are needed to investigate bone loss in these populations.

## 3. Bone Health in the Adult Developmental/Intellectual Disability Population

### 3.1. Low Bone Mass Density Prevalence

It is a consensus that adults with D/ID have low BMD. Most of the available studies do not provide *T* or *z* scores for various age groups, but rather a mean for the entire study population. Other studies meanwhile do not provide *T* or *z* scores at all. In a BMD study of 94 individuals with mild to severe ID (53 females and 41 males, 12 with DS but no gender specified) with a mean age of 35 living in the community, it was found that females possessed a mean *z* score of lumbar sacral spine of −0.6 and males a mean *z* score of −0.4 [22]. Since research of BMD in individuals with D/ID had been restricted to small population sizes, Zylstra et al. conducted a cross-sectional study to investigate the prevalence of osteoporosis in a larger population living in the community [23]. This study comprised 298 individuals (167 males and 131 females) with mild to profound ID aged 6–90 years. The rate of osteoporosis of the femur bone was 17.1% and the rate of osteopenia was 51.0%. Additionally, the mean *T* score was found to be −1.71 for all the ages and both genders. Osteoporosis rates for individuals aged 45 or younger were significantly less than those for individuals aged 46 and older (36.6% versus 48.4%). Although the population size was small in the >65-year-old subgroup, the investigators found that the females in this age group had fewer low BMD scores that met the criteria for osteoporosis than the men. Hence, 3 out of 12 (or 25%) females and 3 out of the 7 males (or 43 %) aged 66 and older met criteria for osteoporosis, which was similar to results reported previously [24]. It was found in the Zylstra [23] study that 19.2% of males with ID had osteoporosis, compared to 14.5% of females with ID. Another study uniquely compared how many individuals carried the diagnosis of osteoporosis prior to a DXA measurement [25]. In this study 107 adults with D/ID aged 40–60 years, living in the community, were investigated. Only 1% of the entire sample had a preexisting diagnosis of osteoporosis and only 4% were taking calcium supplements, while 34% of the subjects were found to be osteopenic and one-fifth of the group (21%) was found to be osteoporotic. A research cohort of 108 institutionalized men (mean age of 52) with intellectual disability, CP, or autism had a mean *T* score of −1.96 and an average *z* score of −1.30. 34% of this study group had a *z* score of ≤−2 below those of age-matched controls. No steady decline of mean *z* scores by age groups was apparent, but rather a variability of scores across ages was observed: 20–29 years: −0.68, 30–39 years: −1.92, 40–49 years: −1.37, 50–59 years: −1.40, 60–69 years: −1.05, 70–79 years: −0.95, and 80+ years: −1.25 [26]. An additional study including both institutionalized men and women with ID found that 28 out of 50 males (mean age of 54) and 32 of 58 females (mean age of 53) had broadband ultrasound attenuation results 2 SD units below the expected mean value for the patients' age [27]. Seven of these individuals were given a DXA and 4 were found to have lumbar spine or femoral neck *T* scores of more than 2.5 SD units below the mean for the same gender, with a trend of lower *T* scores for the males [27]. A larger sample of 562 adults with D/ID living in a long-term care facility from ages 30 to 65+ (mean age 45) had a mean *T* score of −0.8 and mean *z* score of −0.85 for all participants. Out of the 191 males, 10 were older than 65 and 4 (or 40%) had osteoporosis and 1 (or 10%) had osteopenia. Out of 96 females, 5 were older than 65 and 3 (or 60%) had osteoporosis and 1 (or 20%) had osteopenia [28]. In a later study (132 men and 79 women), it was found that more than three quarters (77%) of the study population had a low BMD [28]. In this report the mean age for women was 72.1 years, 73.8 for men aged over 60, and 42.7 years for men under age 60. Of the participants, 35.5% had mild to moderate ID and 64.4% had severe to profound ID. No actual *T* scores were provided, but the authors reported that 62% of participants of 60 years and older had *T* scores of <−2.5 and one-third had *T* scores of −1 to −2.5. Stratified by gender, 67% of women aged 60+ were osteoporotic, compared to 48.5% of men in the same age group. It was found that 26% of men under 60 years were osteoporotic and 36% were osteopenic. Among residents of 60 years and older, women were found to be three times more likely than men to have low BMD [28]. These various cross-sectional studies indicate that adults in the community setting and institutions with D/ID have low BMD. The natural history and mechanisms underlying this condition for this population, however, remain unclear. There is an evident trend of lower BMD in adult males than females with D/ID, although this is not statistically significant and has not been demonstrated in all of the clinical studies.

### 3.2. Low Bone Mass Density Risk Factors

Several risk factors for bone loss and low BMD that have been characterized in the general aging population have also been assessed in people with D/ID. Similar to the Dutch guidelines for the prevention of osteoporosis and falls [17], age [22–24, 28], body weight [22], and immobility status [23, 24, 28] were assessed, and all three of these factors were found to be associated with low BMD. Age greater than 60 [29] and 65 years [23, 24] was associated with lower BMD in both genders, although it has to be pointed out that in the Jaffe and Timell study, only women 60 years and older were included. Similar to the general population, Caucasians with D/ID were found to be at higher risk for low BMD [23, 24]. Additionally, circulating hormone levels were assessed in one study and hypogonadism was found to be associated with low BMD in females [22]. Unique risk factors assessed in the adults with D/ID include phosphate in women [22], degree of intellectual disability [23, 24, 28], antiepileptic drug use [28], DS status [22, 25], and hypothyroidism [25]. All of these unique factors were associated with low BMD in adults with D/ID, except hypothyroidism; however, hypothyroidism was only assessed in one study [25]. Interestingly, the level of ID that was found to be most strongly associated with low BMD was profound ID [23, 24, 28]. 

### 3.3. Bone Mass Density Measurements

The DXA scan is the standard measurement technique of BMD of the hip and lumbar spine in the general population, but in people with D/ID various other techniques and body regions have also been evaluated. These measurements include ultrasounds of the heel [26–28], peripheral DXA of the finger [24], photon absorptiometry of the lumbar sacral spine [22], and central DXA of the femur [23]. These various techniques were not tolerated well in all of the individuals however, due to anatomic deformities or behavioral alterations. For example, 222 of 562 subjects could not have BMD measured due to anatomic deformities or noncooperation [24], and it is likely therefore that different measurement techniques of BMD will be needed in different groups of people with D/ID, depending on their health status.

## 4. Bone Fractures in the Adult D/ID Population

### 4.1. Bone Fracture Prevalence

In addition to low BMD, adults with D/ID are also at risk for bone fractures. As with the normal aging population with a disability, fracture detection can be challenging and can be delayed in this population due to profound cognitive, skeletal, and expressive disabilities that prevent the individual from reporting the fracture event or associated pain [29]. Therefore, when caring for these individuals, joint pain should also be factored into the differential diagnosis if an individual is acting out of character [30]. In one study, it was found that adults with D/ID, who were exhibiting destructive behaviors, demonstrated an increased risk of falls and fractures [31]. While such destructive behavior may have been the cause of the fall and subsequent fracture, it may also have been due to the pain from a fracture. A chart review of 994 residents investigated the fracture rate during a 3.5-year period. In this report 182 bones were fractured, giving a fracture rate of 5.2 fractures per 100 persons/year [32], compared to 3 fractures per 100 persons/year in the US civilian noninstitutionalized population from 1980 to 1981 [33]. A review of accident and radiograph reports and institutional registries of 553 individuals with D/ID were reviewed to determine an accurate fracture rate for adults with D/ID. Fracture rates over a 10-month period were compared to all residents of an institution who did not suffer a fracture during the 10-month study period. The mean age of the case participants was 46 years, versus the control participant mean age of 51 years. It was found that 61 fractures occurred among 55 adult residents with D/ID, giving an annual rate of 13.2 fractures per 100 persons/year in the institutionalized adults with D/ID. Men aged 45 to 64 had a higher fracture rate than those 65 and older and those 44 and younger. Women 65 and older had an increased fracture rate, but the difference was not statistically significant [33].

### 4.2. Fracture Risk Factors

Common fall-related risk factors for fractures in the normal aging population include more than one fall in the past year, the use of psychoactive drugs, a low level of daily living activities before the current fracture, articular symptoms, impaired vision, urinary incontinence, Parkinson's disease [18], and immobility [34]. As with the normal aging population, the level of activity/mobility and antipsychotic medications were risk factors that were assessed in the adults with D/ID. Unique risk factors assessed in the adults with D/ID included level of intellectual disability, antiepileptic use, and epilepsy. Immobility, on the other hand, was found to be a risk factor for recurrent fractures in the general aging population [34], while in the people with D/ID immobility was assessed as a risk factor for first-time fractures. Although not all studies were in agreement [35], overall, the trend was that ambulators seemed to be more at risk for falls than nonambulators [29, 31–33]. For example, the risk of fracture among residents who were independent ambulators was found to be 2.5 times higher than residents who were immobile [33].

### 4.3. Fracture Sites

Specific fracture sites in people with D/ID have been investigated. In one study, fracture data from a 23-year longitudinal cohort registry of 1434 people with severe and profound developmental disabilities identified that 85% of all fractures involved the extremities and the femoral shaft [29]. There are likely to be two general fracture mechanisms in individuals with D/ID: one, which is largely associated with a lack of weight bearing in people with the least mobility, exemplified by femoral fractures during nontraumatic events (e.g., diapering or transfers); the other, probably due to movement- or fall-related trauma, which is exemplified by hand/foot fractures in people who ambulate [29]. A chart review of 994 adult residents with D/ID revealed that falls were related to 41 (23%) of the fractures, and the cause was indeterminable in 105 (58%). It is likely that the indeterminable fractures were due to patient care such as transfers or bathing [29]. Additionally, hand and foot fractures were also found to be common in this population [32, 36]. This was in accordance with a previous study, which showed that 52% of their individuals with D/ID had fractures of the hands and feet (especially for those under age 65) [33]. In contrast, Glick and colleagues (2005) [29] found that femoral shaft fractures decreased with age while hand/foot fractures increased with age [29].

## 5. Prevention and Treatment of Low BMD in**** Adults with D/ID

This section is not intended to be an exhaustive discussion of all the possible prevention and treatment strategies. Rather, the purpose is to introduce a few studies and considerations. A systematic review investigated 6 randomized control trials and 2 controlled clinical trials that investigated the efficacy of various treatments for low BMD in children and adolescents with CP. One of the three bisphosphonate trials showed a large and significant effect on BMD of the femur; one of three weight bearing studies also revealed a large and significant effect but on the lumbar spine [37]. A pilot study demonstrated that 18 months of growth hormone therapy was associated with a statistically significant improvement in spinal BMD [38]. Although numerous studies such as these have investigated treatment of low BMD in children [37–40], relatively few studies have included adults. Since vitamin D levels are essential for normal skeletal mineralization and bone metabolism, vitamin D status was evaluated in institutionalized adults with ID in an open label trial [41]. This study compared oral and intramuscular (IM) administration of vitamin D in 138 Finnish adults with ID. Currently, IM vitamin D is rarely utilized to treat low BMD. Baseline serum 25-OH-vitamin D, calcium, phosphate, alkaline phosphatase, and parathyroid hormone (PTH) levels were measured. The cohort was divided into two treatment groups: one group received vitamin D3 (800 IU) orally per day; the other group received a single vitamin D3 dose (150,000 IU) as an intramuscular injection. All participants were also administered calcium orally (1000 mg per day). Serum 25-OHD levels were measured again at 6 months after the onset of starting the vitamin D and calcium regimen. The gender distribution and proportion of participants on antiepileptics were similar in both groups. None of the participants were taking vitamin D or calcium supplements at baseline, and the majority (65%) of patients were ambulatory. At baseline, there were no significant differences in biochemical values between the two groups, and the means for 25-OH vitamin D were 40 nmol/L and 41 nmol/L, respectively. These low vitamin D levels were associated with secondary hyperparathyroidism in 17% of the patients. In the group that received oral vitamin D, the mean S-25-OHD increased to 82 nmol/L. In the group that received vitamin D through intramuscular injection, the mean S-25-OH increased to 62 nmol/L. The plasma PTH levels decreased in both groups. This study demonstrated that vitamin D insufficiency is common in adults with ID and that either oral or intramuscular administration of vitamin D can increase mean S-25-OHD levels without adverse effects [41].

A treatment study was recently conducted to determine the safety and efficacy of teriparatide (a recombinant form of PTH) in nonambulatory institutionalized men and women with osteoporosis [42]. In this trial, bone biomarkers were used to assess efficacy. Teriparatide (20 *μ*g subcutaneous) was administered daily for up to 18 months at one institute and 24 months at the other [42]. All participants received at least 400 IU vitamin D orally a day and at least 1000 mg/day of calcium. Markers of bone formation (procollagen type 1 intact N-terminal propeptide (PINP)) and resorption (C-telopeptide (CTx)) were measured at 3-month intervals. Serum calcium was measured at 2 week intervals for 12 weeks and thereafter at 3-month intervals, and 27 individuals received at least one injection. The incidence of hypercalcemia was 11.1% and led to medication discontinuation in one participant. Biomarkers of bone formation increased, doubling by three months. At 12 months, PINP and CTx levels remained elevated from baseline. It was concluded that teriparatide was safe and effective in this population, however serial calcium measurements are recommended, especially during the first 3 months. It has been suggested that bisphosphonates necessitate maintaining an upright posture for at least 30 minutes after taking them, which may be difficult for some D/ID individuals [31]. Also, the risk of esophageal ulceration may be increased in individuals with D/ID whose disabilities include oral motor dysfunction, which is particularly a concern if the individual is unable to communicate the pain [31]. 

## 6. BMD and Down Syndrome (DS) 

DS, trisomy of chromosome 21, is the most common identified cause of severe intellectual disability. In addition to intellectual disability, it is associated with cardiac, endocrine, gastrointestinal, skin, hearing, and vision dysfunction, and growth failure [43]. The Centers for Disease Control and Prevention (CDC) estimates that each year approximately 6,000 babies in the United States are born with Down syndrome (1 of every 691 births) [1]. In order to try to understand the generation of abnormal BMD in the DS and CP populations, studies that include children will also be included in our paper. In a small study, the BMD of 10 Chinese children with DS (7 boys, 3 girls, aged 10–16 years) was compared to 10 age-matched controls. The BMD of the 2nd to 4th lumbar vertebrae was measured using a dual photon absorptiometer. The BMD of the DS patients' values ranged from 0.65 gc-2 in the 10-year olds to 1.00 gc-2 in the 16-year olds and was reportedly lower than the controls. The BMD of the DS children increased from age 10 to 16 years, but was reportedly significantly lower than in normal children of the same age group [44]. A more recent cross-sectional study [45] utilized a larger sample size of children and adolescents with DS and corroborated the results by Kao et al. [44]. Thirty-two children and adolescents (15 females and 17 males) with DS between 10 and 19 years were compared to an age-matched sample of 32 healthy subjects (13 females, 19 males) without DS. Bone mass at the lumbar spine (L1–L4) and femur (hip and femoral neck) was measured with DXA using a pediatric version of the software. Volumetric BMD (vBMD) was estimated for the lumbar spine and femoral neck using simple geometric cylindrical models. BMD/height (BMDH) was calculated to adjust bone mass for whole body bone size. No results were provided for specific ages within this cohort, however. After adjusting the raw values by Tanner stage (a scale used to describe the onset and progression of pubertal changes) height, and total lean mass, it was found that females with DS, compared to females without DS, had lower bone mass density (0.085 ± 0.008 versus 0.092 ± 0.004) and BMD/height (0.61 ± 0.04 versus 0.64 ± 0.01). DS males and females had lower BMD in the whole body compared to the controls (males: 0.928 ± 0.127 DS versus 1.049 ± 0.128 controls, females: 0.845 ± 0.086 DS versus 1.014 ± 0.109 controls). DS females had lower lumbar spine BMD than the DS males (0.76 ± 0.118 versus 0.788 ± 0.146) [45]. Baptista and colleagues also compared bone mineral mass adjusted for bone and body size in limbs, lumbar spine, and the femoral neck but did not find significant findings in their subjects that were less than 20 years [46]. Their subjects consisted of 66 females (33 with DS) and 68 males (34 with DS) aged 14–40 years, living in the community. DXA was used to measure BMD, and volumetric BMD and femoral neck strength were calculated. DS was shown to be a risk factor for both low lumbar spine volumetric bone mineral density and femoral neck strength in the group older than 20 years old, compared to adolescents. In another study, Guijarro and colleagues also investigated the impact of short stature of DS patients on bone mass [47]. Their assessment involved 39 ambulatory patients with DS (18 male, 21 female) ranging in age from 18 to 45 (mean age of 26) and age-matched controls. A DXA was used to assess BMD of the spine, hip, and the total body and percentage of fat and lean mass, and the volume of BMD at the lumbar spine and femoral neck was computed. A reduced BMD was found in all DS patients in the spine, hip, and total body. Spine volume BMD was also lower in DS than controls (0.140 versus 0.149 g/cm^2^) [47]. The main causes of low BMD in the DS population are likely to include endocrine abnormalities such as thyroid and gonadal dysfunction, reduced physical activity, hypotonia (low muscle tone), and reduced muscle strength [22, 47, 48]. It has also been suggested that the distal region of chromosome 21 may be associated with osteoporosis [49]. Although men with ID with and without DS have been shown to have lower quadriceps muscle strength, only DS males demonstrated lower spine BMD than the healthy males of the control group [48]. The limited studies to date suggest that although DS children as young as ten years old have abnormal BMD it is not until after about age 18 that the BMD was found to decrease. However, limited *T* and *z* scores were included in the results. 

## 7. BMD and Cerebral Palsy (CP)

CP is the most common motor disability in childhood, and the CDC estimates that an average of 1 in 303 children in the USA are diagnosed with CP [1]. CP consists of a group of permanent disorders of movement and posture development, causing activity limitations, which are attributed to nonprogressive disturbances that occurred in the developing fetal or infant brain. The motor disorders of CP are often accompanied by disturbances in sensation, perception, cognition, communication, and behavior, by epilepsy and by secondary musculoskeletal problems. The range of movement limitation is variable and may range from independent ambulation to an inability to raise one's head. A more detailed discussion of the classification of cerebral palsy was recently reviewed [50]. 

Over the past thirty years, various studies have investigated BMD and fractures in children with moderate to severe CP. Recently, a systematic review was completed that identified a limited amount of high-quality evidence on low BMD and fractures in children with severe CP [51]. Fractures are common in individuals with moderate to severe CP, with an incidence of approximately 4% per year [52], compared to approximately 2.5% in healthy children [51]. Low BMD is a serious problem in children with moderate to severe CP. Clinical *z* scores have been found to range from −3.4 in the distal femur to −0.8 in the lumbar spine [53–55]. There has also been shown to be a relationship between advancing age and declining BMD *z *scores at distal femur sites. Ages 2 to 5.9 had a *z* score of −2.9 ± 0.4, ages 6–11.9 had a *z* score of −3.0 ± 0.2, and ages 12 to 19 had a *z* score of −3.7 ± 0.3. It was found that 71 boys had a mean *z* score of −3.1 ± 0.2 of the distal femur and 46 females had a mean *z* score of −3.6 ± 0.3 of the distal femur [53]. These similar *z* scores of the distal femur of the same age groups were also replicated in a more recent study by Henderson and colleagues [55]. Additionally, the BMD of the radius and tibia was measured in 45 children and young adults with moderate to severe CP by ultrasound. The *z* scores for the radius were from ages 1 to 10 −0.8 ± 1.1, ages 11–20 −1.0 ± 1.4, and ages 21–29 −1.1 ± 1.1. The females had a mean *z* score of −1.4 ± 1.3 and males −0.6 ± 1.1 [56]. The former two studies [53, 54] measured the BMD of the femur, which is the most commonly fractured in this population, with a DXA; the latter study on the other hand used ultrasound to measure the radius. The prevalence of low BMD of the distal femur measured by DXA (defined as *z* score lower than −2) was 77% in a population of 117 children aged 2–19 with moderate to severe CP. The most commonly studied determinants of low BMD were gross motor function classification system (GMFCS) level, feeding difficulties, previous fracture [51, 53], and the use of antiepileptic drugs [57, 58]. Severity of neurologic impairment measured by GMFCS, increasing difficulty feeding the child, use of antiepileptics, and lower triceps skinfold all independently contributed to lower BMD *z* scores in the femur (in decreasing order of importance) [53]. It was accepted that absence of weight bearing is an important direct cause of low BMD in children with CP; however, it was found that BMD *z* scores were significantly lower in GMFCS level 5 children than in level 4 children, yet both groups are nonambulatory [53]. Based on these studies, there seems to be a trend towards increasing age and low BMD in CP. Uniquely, the low BMD has been found in children as young as age 2.

Anthropometric and fitness variables were also recently assessed to determine if they would be useful for detecting children with potentially reduced bone density. Growth variables were mainly related to femoral and lumbar bone densities, while muscular endurance was mainly related to femoral and calcaneus bone densities. This suggests that multiple complex variables can contribute to bone density variations among different skeletal areas in these children [59]. Results from another recent study suggest that muscle strength, especially antigravity muscle strength, was more associated with the bone density of ambulatory children with CP than motor function [60]. However, it must be noted that not all predictive factors for developing low BMD in this population have been studied; for example, daylight exposure time and amount of exercise have not been investigated [51].

A limited number of studies have investigated the bone health of adults with CP [57, 61]. In the Nakano et al. study, 123 institutionalized adults with CP (mean age of men was 31.5 years and mean age of women was 33.3 years) had the BMD of their 2nd metacarpal bone measured by a hand absorptiometer [61]. Although no actual *T* or *z* scores were provided in this study, the authors reported that the study subjects had poor bone health. Ambulation was significantly associated with higher BMD in women, and hypocalcaemia, hypophosphatemia, and elevated alkaline phosphatase levels were found in 28% of the men and 31% of the women [61]. In the King et al. study [57], it was investigated whether children and adults with spastic quadriplegic CP, who are nonambulatory, would have lower BMD that worsens with age when compared to age- and gender-matched controls. In this study 51 participants were recruited from institutions and community settings. BMD was measured with DXA, skeletal surveys were performed to assess fractures, and biochemical analyses including calcium, phosphorous, magnesium, 25-OH vitamin D, and osteocalcin were performed. Participants ranged from 5 to 48 years of age. The mean *z* score for the lumbar spine for all study participants was −2.37 ± 0.21. When the participants were divided into groups of 18 years or less and greater than 18 years, the *z* scores were similar; for those 18 years or less the mean *z* score was −2.32 ± 0.23 gm/cm^2^ (*n* = 30) versus −2.45 ± 0.40 for those greater than age 18 years. Interestingly, no correlation was found between BMD *z* score and age in this study. There were no differences in BMD *z* score between all participants when corrected for bone age versus uncorrected [57]. 

## 8. Conclusions

Many studies have demonstrated that BMD is decreased in both men and women with D/ID, and especially those with DS and CP. Some of the risk factors for decreased BMD in D/ID include endocrine abnormalities such as thyroid and gonadal dysfunction, reduced physical activity, hypotonia and reduced muscle strength, vitamin D deficiency, and certain medications. It is presently unclear whether the bone mass decrease is similar to that seen in populations without D/ID or whether it is the continuation of a process begun in early childhood, and future studies will be needed to address this important topic. The DXA scan is currently the standard measurement technique of BMD of the hip and lumbar spine in the general population, but in the D/ID population these specific measurements are not always feasible to collect. Thus, it is also clear that one size does not fit all with regard to measurement techniques of BMD in people with D/ID and future studies are needed that thoughtfully and creatively identify appropriate tools for measuring the BMD in this population. Measurements taking into account body and bone size (by calculating an adjusted BMD) as like was done for individuals with DS may need to be replicated for other syndromes or endocrine diagnosis that include short stature such as Turner syndrome, Rett syndrome, or growth deficiency.

Although various genetic and environmental risk factors for low BMD in individuals with D/ID have been identified, the exact molecular mechanisms underlying the low bone density have yet to be elucidated. Until the mechanisms for this diverse population are identified it is difficult to begin to propose logical nonpharmacologic or pharmacologic interventions. One low BMD prevention study and one treatment study for this population utilized serum biomarkers as an outcome marker. It would be practical and clinically relevant to take the outcome measurement a step further and to investigate improved repeat BMD and/or overall reduction in fractures. Independent of low BMD, clearly this population is at risk for fractures. The majority of the studies were in agreement that the fracture rate increased with mobility and that hand and feet fractures were the most common, although a large proportion of the populations had fractures without a known cause. This paper has specifically focused on discussing D/ID, DS, and CP in relation to BMD. However, BMD is only one measure that can be used to assess a very complex structure such as bone. Several studies have found differences in other bone health variables in genetic syndromes [62, 63], and children with CP (even as young as 2–5 years old) have been found to have low BMD. This raises the question of the direct effects of gene mutations or polymorphisms on bone cellular processes [64] or secondary effects due to neurological or structural impairments that affect bone health [62]. For example, individuals with the developmental disorder Prader-Willi (PW) syndrome are known to develop osteoporosis [65]. This is thought to be caused by an abnormal expression of genes within the PW-critical region on bone cellular functions and is secondary to associated findings, such as hormonal imbalances and hypotonia [62]. It is clear that further work is needed to unravel these complex findings, and gaining a greater understanding of changes in BMD in people with D/ID could lead to the development of more advanced diagnostics and treatments for bone loss in this population.

## Abbreviations

ID: Intellectual disability
DD: Developmental disability
DS: Down syndrome
CP: Cerebral palsy
BMD: Bone mass density
vBMD: Volumetric bone mass density
DXA: Dual-emission X-ray absorptiometry
US: Ultrasound
S-25-OH: Serum 25 hydroxyvitamin D
PTH: Parathyroid hormone
CDC: Centers for Disease Control and Prevention.
